# Tail-Biting in Pigs: A Scoping Review

**DOI:** 10.3390/ani11072002

**Published:** 2021-07-05

**Authors:** Maggie Henry, Hannah Jansen, Maria del Rocio Amezcua, Terri L. O’Sullivan, Lee Niel, Anna Kate Shoveller, Robert M. Friendship

**Affiliations:** 1 Department of Population Medicine, University of Guelph, Guelph, ON N1G 2W1, Canada; Mhenry12@uoguelph.ca (M.H.); hjansen@uoguelph.ca (H.J.); mamezcua@uoguelph.ca (M.d.R.A.); tosulliv@uoguelph.ca (T.L.O.); niell@uoguelph.ca (L.N.); 2 Department of Animal Biosciences, University of Guelph, Guelph, ON N1G2W1, Canada; ashovell@uoguelph.ca

**Keywords:** swine, tail-biting, risk factors, abnormal behavior, intervention strategies

## Abstract

**Simple Summary:**

Tail-biting in commercial pig-rearing facilities is a welfare concern. Serious outbreaks may occur sporadically, particularly in the grower-finisher stage of production. A scoping review was undertaken to determine if there is general agreement on the common risk factors and appropriate intervention strategies of tail-biting. Online databases were explored for relevant articles and information was extracted from the articles based on predetermined questions. Important risk factors varied between studies, as did successful interventions. The provision of rooting material to alleviate a pig’s innate exploratory behavior was determined to be a common prevention/intervention strategy. Deciphering between risk factors and interventions in the literature was not always possible. Overall, the cause of tail-biting was determined to be multifactorial.

**Abstract:**

Tail-biting is globally recognized as a welfare concern for commercial swine production. Substantial research has been undertaken to identify risk factors and intervention methods to decrease and understand this vice. Tail-biting appears to be multifactorial and has proven difficult to predict and control. The primary objective of the scoping review was to identify and chart all available literature on the risk factors and interventions associated with tail-biting in pigs. A secondary objective was to identify gaps in the literature and identify the relevance for a systematic review. An online literature search of four databases, encompassing English, peer-reviewed and grey literature published from 1 January 1970 to 31 May 2019, was conducted. Relevance screening and charting of included articles were performed by two independent reviewers. A total of 465 citations were returned from the search strategy. Full-text screening was conducted on 118 articles, with 18 being excluded in the final stage. Interventions, possible risk factors, as well as successful and unsuccessful outcomes were important components of the scoping review. The risk factors and interventions pertaining to tail-biting were inconsistent, demonstrating the difficulty of inducing tail-biting in an experimental environment and the need for standardizing terms related to the behavior.

## 1. Introduction

Tail-biting (TB) is a common problem on commercial swine farms worldwide. Tail-biting is the oral manipulation of a pig’s tail by another pig that causes tissue damage. This abnormal behavior can result in severe injury to the individual pig and have significant economic consequences resulting in an overall reduction in farm productivity. Economic implications for TB include both direct and indirect costs [1,2,3], such as increased labor, increased medical care and amplified condemnations of the carcass at slaughter. The economic cost of TB to pork industries around the world is millions of dollars annually [4]. D’Eath et al. (2016) [2] hypothesized that any occurrences of TB decreased the net profit margin by $23.00 USD/victim pig. Three different subsets of TB have been hypothesized, with various incentives for each type [5]. The first subset is referred to as two-stage TB. Two-stage TB begins with a pre-injury stage and is then followed by visible tail injuries; this form of TB is believed to be instigated by a lack of rooting substrate or a lack of biologically relevant enrichment [5]. The second subset is sudden forceful TB and is characterized by an acute clinical onset of injury with no discernable pre-injury phase. Sudden forceful TB is believed to be precipitated by animal frustration due to a lack of environmental resources and physical discomfort [5,6]. The third and final subset of TB is referred to as obsessive, where the pig becomes obsessed with manipulating conspecifics’ tails. There is no known motivation for obsessive TB; however, it has been hypothesized that it may be due to an underlying genetic component [5]. Research on TB has been conducted over decades, yet the issue persists. Major risk factors for TB and successful intervention strategies have been identified in research trials, but results have not been applied in the field. For these reasons, a scoping review was conducted to chart the available literature and determine if a consensus on risk factors and successful intervention strategies exist, and if not, to discover existing gaps in the literature. The following research questions were formulated: What are the main risk factors for tail-biting in pigs? Which intervention strategies have been researched after 1970? Additionally, what are the best intervention strategies to treat and prevent tail-biting?

## 2. Materials and Methods

Protocol: Scoping review methods were followed as described by Arksey and O’Malley (2005) [7]. The development team included professionals with knowledge of swine research, ethology, nutrition, and veterinary medicine. The protocol was constructed a priori and was published on the University of Guelph Atrium repository [8]. The initial protocol as well as revisions made to the protocol (Final Protocol) are available in Supplementary Material S1. The purpose of this scoping review was to gain insight into which risk factors were considered important to the origin of TB behavior in pigs and to identify the intervention methods which had been implemented and demonstrated to be effective against this behavior.

Eligibility Criteria: In most areas of the world, intensive rearing of swine began after 1960 [9,10], and research pertaining to behavioral issues of these intensively raised pigs was not conducted until the 1970s [9,11]. With this knowledge, an exclusion for any articles prior to 1970 was created. All English-language articles published between 1 January 1970 and 31 May 2019 were acceptable for inclusion by the two reviewers. The two reviewers spoke English as their primary language, and it was decided that only articles originally published in English or translated and available in English would be included in the scoping review. Literature was not excluded based on geographic location, study design, stage of production, production type (intensive or extensive) or population (research or commercial).

Information Sources: Three databases—CAB Direct, Web of Science and AGRICOLA—were searched for peer-reviewed literature. The American Association of Swine Veterinarians’ Online Information Library (AASV) was searched for grey literature.

Search: The search string for all databases (CAB Direct, Web of Science, AGRICOLA and AASV) was identical. Search terms (SWINE, RISK, TAIL) were decided upon by identifying the relevant terms in the review question and by examining whether these terms captured variations in descriptive words that would be applicable to the scoping review. For example, SWINE was used as a search term to verify that articles containing “pig”, “pigs”, “pork” and “*Sus scrofa*” were all returned by the search term. It was concluded that SWINE was acceptable as an agreed upon term for the scoping review. RISK and TAIL were both included in the search terms with an “*” at the terminus of each word to capture any word (s) beginning with the root word “risk” or “tail”, for example risks, tails, risk factors, and tail-biting. The Boolean term AND was used between the three words. The search headings for each database were as follows: SWINE AND TAIL * AND RISK *.

Selection of sources of evidence (relevance screening) and data charting process: Literature returned from the database search was downloaded to Zotero reference library (© 2019 Corporation for Digital Scholarships) for Level 1 (L1) screening and duplication detection. It was agreed that all returned literature from the initial search which passed inclusion criteria would have the reference list searched to include any literature not identified by the search string but relevant to the research questions. Level 1 screening consisted of title and abstract screening to assess the relevance of the article to the research question of the scoping review. Upon completion of L1 screening, the full texts of all articles were obtained using The University of Guelph Library’s access to literature databases, and librarian-assisted retrieval for documents that were not available online. All agreed upon articles, based on inclusion and exclusion criteria (see Protocol), were then uploaded to Distiller SR (© 2019 Evidence Partners Inc.) for Level 2 (L2) screening and data charting. Duplication detection was once again completed on all uploaded documents. Level 2 screening entailed reading of the full text and charting the information in each article using the data charting form specifically designed for the current scoping review. Two independent reviewers completed both the L1 and L2 screening processes and disagreements about inclusion of articles were resolved by consensus. Throughout L1 screening, any article that was not a clear exclusion candidate was included in L2 screening as a means of decreasing uncertainty at the abstract level. A final list of included articles is available in Supplementary Material S2.

Synthesis of Results: All data were downloaded into Microsoft Excel for curation and then imported into Stata (StataCorp. 2019. Stata Statistical Software: Release 16. College Station, TX, USA: StataCorp LLC) for analysis. Frequency tables were created to determine comparisons between charted variables.

## 3. Results

**Figure 1:**
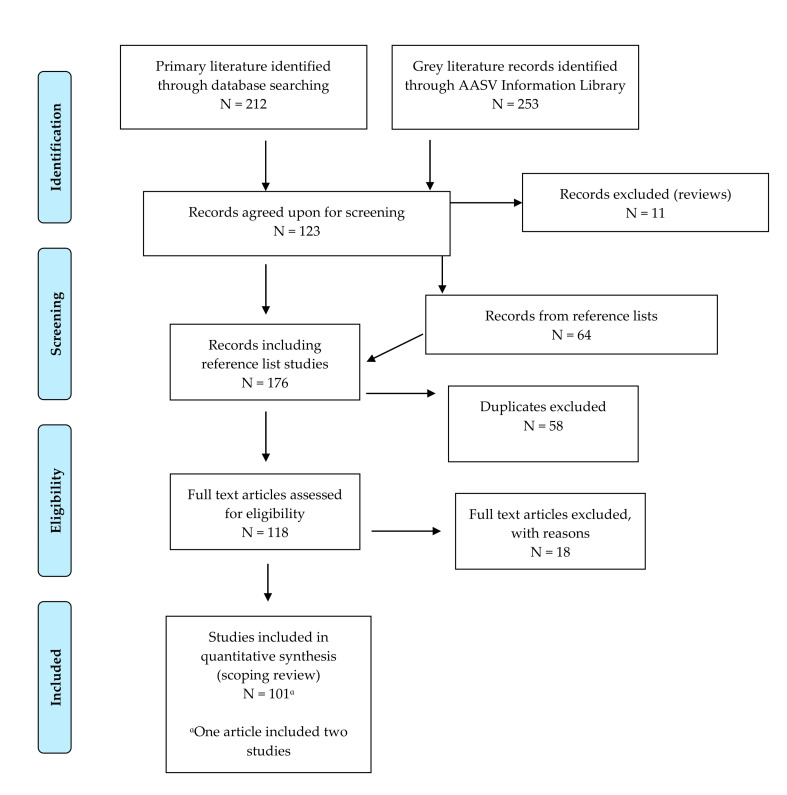
Preferred Reporting Items for Systematic Reviews and Meta-Analyses (PRISMA) flow diagram of included literature in the scoping review process.

Results of the database searches are summarized in the Preferred Reporting Items for Systematic Reviews and Meta-Analyses (PRISMA) flow diagram (Figure 1). The search yielded a total of 465 unique citations from both the primary and grey literature databases. Of the citations returned, 342 were excluded as non-relevant, based on their titles and abstracts, leaving a total of 123 citations. Of the 123 articles, 11 were deemed ineligible, as they were reviews, rather than original research. The reference lists from the 112 accepted articles yielded 64 new articles that had not been screened by the reviewers. After screening the 176 accepted articles for duplicates, 58 articles were excluded, leaving 118 articles eligible for L2 screening. One hundred full-text articles (101 studies) met the inclusion criteria and were charted for quantitative synthesis.

Articles in the final scoping review included 93 full-text primary research journal articles, seven research reports and one conference proceeding. Clinical trials were the most reported study type (61/101; 60.4%), followed by observational studies (38/101; 37.6%), while there was insufficient information to classify two of the articles into a study type. The most common observational studies included 14 case–control studies, 11 cross-sectional studies and 7 cohort studies. 

**Figure 2:**
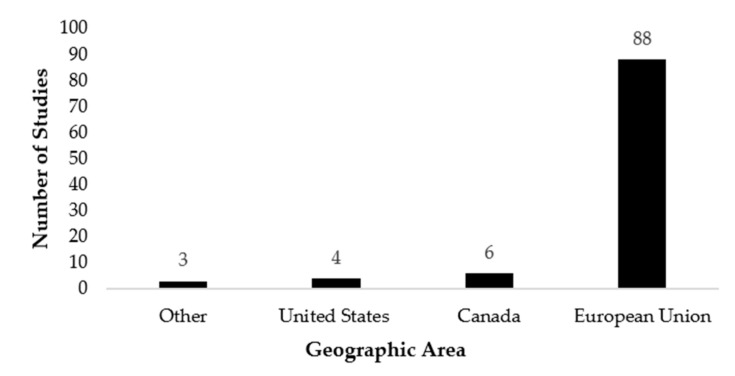
The geographic area of where each study included in the scoping review was conducted. For studies not listing a country of origin, the first author’s affiliations were used as study location.

Publications from each of the five decades (1970s, 1980s, 1990s, 2000s, and 2010s) included in the search strategy were accepted for the review, with 87/101 (86.1%) studies published in the period 2000–2019. The majority of the articles included in the scoping review involved studies conducted in the European Union (EU) (88/101; 87.1%), while six studies were performed in Canada, four studies in the United States (US) and three studies from other countries (China, Australia and Brazil) (Figure 2).

Reported population parameters were discovered to be mainly commercial farms (54), while research populations (37) were the second most common population. No population type was reported in nine of the total articles, and one article reported using both a research and a commercial population for their study.

**Figure 3:**
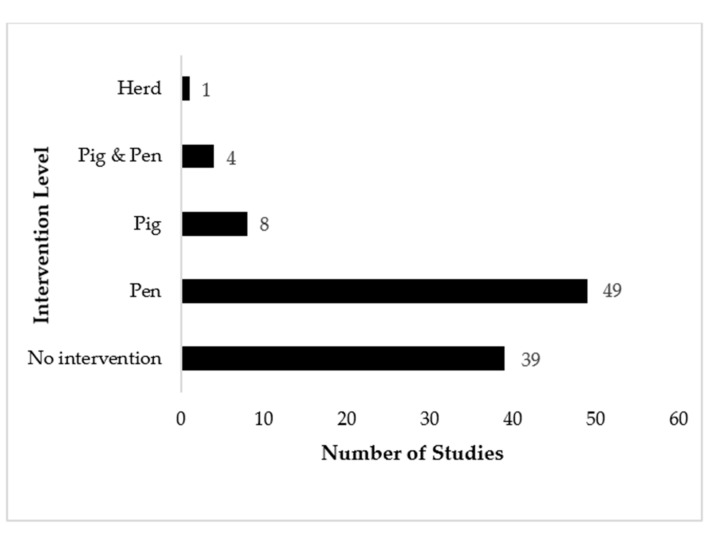
Number of studies which included interventions and the level at which the intervention was applied.

Intervention strategies applied to a population ranged from no intervention to interventions at the individual pig level, the pen level and the herd level, with those at the pen level being the most common (49/101; 48.5%). No intervention was reported in 39/101 (38.6%) of the studies (Figure 3). Of the articles reporting an intervention, 37/62 (59.7%) studies reported a successful intervention outcome, while 25/62 (40.3%) studies reported an unsuccessful intervention outcome after the intervention had been applied (Table 1). Stage of production at the beginning of the study and at the termination of the study varied (Table 2). Grower-finisher pigs were the most common stage of production at the commencement of a trial, while finisher pigs were the most common stage of production at the conclusion of the study.

Outcomes measured in the studies did not exclusively pertain to TB. The most common outcome measured was TB (88/189; 46.6%); however, production accounted for 41/189 (21.7%) outcomes and other accounted for 46/189 (24.3%) outcomes (Table 3). Reported outcomes for TB included lesion frequencies, tail length or tail damage, as well as condemnations at slaughter. Lesion frequency and tail length/damage were the most reported TB outcomes, accounting for 59/174 (33.9%) and 77/174 (44.3%) outcomes, respectively (Table 4).

Several risk factors were investigated in the included studies. The most reported risk factor was enrichment, followed by housing or flooring type (Table 5). The most common intervention method investigated was straw or other rooting substrates, while the most successful intervention method was tail docking (Table 6).

Important potential gaps in the research: The limited amount of research concerning TB from outside of Europe within the last decade is a visible gap identified in this review. Swine rearing systems differ according to geographical location due to legislation, genetics, nutritional components of the diet and export markets; however, the lack of research from North America and Asia regarding an ongoing pig welfare issue may invite criticism of these pig industries. The limited quantity of research pertaining to genetics and the possibility of decreasing TB through knowledge as to which breeds, or genetic lines are more inclined to bite conspecifics is also a significant gap in the literature. Several risk factors and interventions were explored in the scoping review; however, identifying the most appropriate stage of production to apply these treatments was inconclusive and warrants further investigation. Rooting material was identified as a successful intervention strategy, yet the timing and quantity of when rooting material was offered differed according to study design and was not reported consistently. The limited amount of research investigating rooting substrate which is also compatible with liquid manure management systems is a gap in TB research.

## 4. Discussion

An objective of this scoping review was to identify the main risk factors for TB behavior. Several risk factors were identified in multiple papers but there was conflicting evidence regarding the relative importance of each of these factors [12,13]. There were also various study methods used to determine risk factors, as some studies used rope and chew tests as tail models [14,15,16] rather than observing pigs. The most prevalent and consistent risk factor outlined in the charted data was a lack of environmental enrichment. Several other risk factors were presented in the literature, although the importance and success of correcting these risk factors were inconsistent between studies [17,18,19]. Studies have demonstrated that TB occurs most commonly during the grower-finisher phase of production [20,21]; however, this review contained studies which included all stages of production, from breeding age pigs to nursing piglets. Investigation into whether risk factors are consistent over time, or if different risks pose greater challenges at specific phases of production may be an area of study which will prove useful in increasing our basic understanding of TB and decreasing the inconsistencies of study results.

Classifying risks and interventions as unique factors was problematic during analysis, as several studies combined or did not explicitly state which measures were risk factors and which were intervention strategies. Intervention studies have revealed inconsistencies as to the success of the various methods commonly employed on commercial farms. The use of environmental enrichment, including rooting material such as straw, typically decreases TB [22,23]; however, the success of a rooting substrate was found to be dependent upon the quantity of material and the general acceptance of the rooting material by the pigs [24,25]. Furthermore, the addition of rooting material relies on the manure management system; unfortunately, the majority of swine farms use liquid manure systems which may be ill-equipped to handle straw [26], or other types of rooting material. This scoping review highlights that further investigation regarding species relevant enrichment which can be utilized with current manure systems is necessary. 

The lack of literature from countries outside of the EU from the past decade was an interesting and concerning finding in this review. Genetics, pig-rearing practices and industry standards have evolved to reflect the current potential of the pigs and the market. Although similar rearing practices are implemented in North America and the EU, and similar quantities of pork and hogs [12,13] are produced in the EU and the US, individual variations within the regulations regarding animal production and animal welfare among the countries represented in the review exist [6,27,28,29,30,31,32,33], and therefore, necessitate original research from all geographic locations. The lack of TB research, outside of the EU, is particularly concerning, as three of the world’s top four pig producing areas: China, the world’s leader in pig production, the US and Brazil [34] only had three publications between them in the last decade, compared to 53 publications from the EU. Pig welfare is becoming more common within China, yet the majority of this work is unavailable in English, as reviewed by Sinclair et al. in 2020 [35], which may have resulted in a perceived lack of data. This apparent lack of research because of limiting this scoping review to English publications may also extend to other non-English speaking pig producing nations. Concern over livestock welfare has been present in the EU for many decades resulting in legislation affecting pig production and animal welfare [4,9,10,11,28,36], and therefore research designed to help guide government policy is likely partly responsible for the large number of publications from the EU. Considering the economic [2,37] and welfare implications associated with TB [2,28], this review demonstrated that there is need for studies relating to TB in pig producing countries outside of the EU to inform modern pig production.

A ban on the tail-docking of pigs has been implemented in the EU [38] due to the negative welfare concerns surrounding the practice and the implication that the procedure does not address the underlying cause of TB, only the visible results; yet, several member states continue to practice this management procedure [4,38,39]. Tail-docking as a routine preventative measure is only acceptable in the EU when TB is present on-farm and all other avenues for decreasing TB behavior have been unsuccessful [4,38,39]. Tail-docking is still permissible and regularly performed on pig farms in North America. Several studies have highlighted the success of tail-docking as a control for TB [12,13,17,22,40]; however, it is known that tail-docking is a painful procedure [41]. Canada has recently required the use of analgesics during tail-docking [30] to diminish post-surgical pain and inflammation. Studies suggest that pigs which are not tail-docked are more susceptible to receiving a tail injury, lowering the pigs’ welfare due to tail damage [41]. Producers have expressed concern over the alternatives to tail-docking, as they may be difficult to implement in current housing environments [3,4,38,39,40].

The majority of studies in this scoping review explored external influences and how they relate to TB occurrences, yet few studies explored genetic predispositions [42,43,44] or nutrition [15] as a precursor for TB behavior. Knowledge of specific genes, such as PDK4, which has been shown to be expressed differently in biter and victim pigs compared to neutral pigs (neither receiving nor performing biting behavior) [44], could encourage genetic companies to purposively select pigs which exhibit a more desirable gene expression. Commercially desirable production traits, such as backfat thickness, have been negatively correlated with TB [43]; therefore it is possible that selection for commercially acceptable and preferred pork products has inadvertently selected for pigs more likely to perform abnormal behavior. It is possible that focusing on specific genetic selection would only address the outcome of TB, rather than the causes of the behavior, similar to the arguments of tail-docking mentioned earlier. However, gene expression can be altered, based on environmental stimuli, such as stress, once again demonstrating the multifactorial nature of TB.

Several of the studies included in the review have examined housing and management; however, research describing the use of nutrition, such as tryptophan, or other feed factors which promote calm pigs were far fewer. Tryptophan is an indispensable amino acid for pigs, as it cannot be produced through metabolic pathways, and can only be obtained through the diet [45,46]. Tryptophan is a precursor for serotonin [45], which is known to increase positive feelings, have a positive effect on feed intake [46] and improve intestinal health [47] in mammals. Feeding high levels of tryptophan as a therapeutic agent has been shown to improve recovery time in pigs experiencing stress [47,48,49]. The small number of studies, relating to genetics and nutrition demonstrates a need for research which explores internal influences of TB behavior. Internal influences may provide an amplified, positive contribution to the study of abnormal behavior, rather than only focusing on the external elements of pig rearing.

The results of this scoping review suggest that the quantity of evidence-based data available to guide decision-making processes regarding risk factors and appropriate intervention methods for decreasing or eliminating TB is insufficient. The overarching factor of management appears to be an important risk factor; however, management is a broad category and deciphering the specific risks within this category are difficult. The origins associated with TB suggest that it is a multifactorial condition [50], and thus, risk factors in general may be hypothesized, yet cause and effect remain difficult to define and possibly specific to each individual farm [51]. The assumed triggering factor, which is believed to initiate TB, once removed, does not guarantee that the problem will cease [50], creating a challenging environment to control the abnormal behavior once it begins. When accepted interventions and known risk factors are addressed, TB may persist [2,52,53,54,55,56]. All aspects of commercial swine production, both internal and external influences, are necessary to investigate when considering the causes and remedies for TB behavior. Multidisciplinary research may be required to truly understand this abnormal behavior. In all likelihood, a factor that clearly triggers a TB outbreak in one situation, may not cause TB when circumstances are altered. The multifactorial nature of the problem makes the study of risk factors and the evaluation of preventative strategies very difficult.

A concern that was revealed by the scoping review is that investigators were sometimes inconsistent in defining TB. Some studies [57,58,59,60,61] included all tail-in-mouth events in their studies of risk factors or prevention strategies and other researchers used models such as pigs chewing on ropes [14,15,16,62,63]. It remains unclear whether conclusions from these studies are equally valid to studies that define TB as aggressive chewing of the tail causing injury. Standardizing the definition of TB and designing studies that can control for the many possible contributing factors are challenges that need to be addressed in future studies.

Future Considerations and Next Steps: The current literature yielded by the scoping review indicates that a systematic review is possible at this time. A systematic review and meta-analysis exploring the most effective risk factors and interventions at each stage of production would aid producers and industry in management and housing legislation in the future. Exploration of study designs used in intervention experiments would be useful to evaluate the significance of each treatment within a research or commercial population to guide recommendations for producers. Ongoing evidence-based research with standardized definitions of TB, pertinent risk factors and interventions at separate stages of production, and further exploration regarding the genetic elements that may contribute to TB will allow further insight into the impacts of direct and indirect costs to the producer.

Limitations: Journal articles available in English were used in this review. It is possible that relevant articles available in other languages were excluded based on language requirements. 

## 5. Conclusions

There is a considerable amount of research which focuses on TB; however, categorizing the most important risk factors and interventions to decrease this abnormal behavior is inconclusive. This review demonstrated that the amount of research concerning TB has increased over the past two decades, particularly in the EU. Although this does appear to indicate that there is interest and willingness to understand TB, this abnormal behavior is under-represented in the literature in three of the four largest global pig producing locations. The main risk factors associated with an outbreak of TB, appear to be both internal and external factors involved in commercial pig production, including; genetics, nutrition, and the absence of species relevant enrichment. Tail-docking, and providing environmental enrichment were shown to be often but not always successful intervention strategies for limiting or decreasing TB behavior, although the timing of the interventions was not always explicitly stated, creating challenges for reproducibility of the studies. Available, consistent evidence-based literature is not definitive, and a more rigorous investigation of study design and significant housing and management strategies related to stage of production is necessary. A major inconsistency among TB research is the definition of the behavior being studied, that is whether TB includes all tail-in-mouth activity or whether it is restricted to chewing on tails to create a wound. 

## Figures and Tables

**Table 1:** Article type including an intervention used and intervention outcome based on stated objective in the study.

	Intervention Outcome		
	Successful	Unsuccessful	Total
Article Type			
Conference Proceedings	1	0	1
Full-Text Articles ^1^	35	23	58
Research Reports ^2^	1	2	3
Total	37	25	62

^1^ Full-text articles included journal articles found in peer-reviewed sources and included an abstract, introduction, methods, results, discussion and reference lists. ^2^ Research reports included short communications which did not meet full-text article inclusion criteria.

**Table 2:** Article type and stage of production listed in the article at the beginning of the study and at the conclusion of the study.

	Stage of Production at the Beginning of the Study					Stage of Production at the End of the Study				
	Nursing	Nursery	Grower	Finisher	Adult	Nursing	Nursery	Grower	Finisher	Adult
Article Type										
Conference Proceedings	0	0	1	0	0	0	0	0	1	0
Full Text Articles ^1^	16	25	18	30	4	0	14	7	67	5
Research Reports ^2^	0	3	2	1	1	0	0	3	3	1
Total	16	28	21	31	5	0	14	10	71	6

^1^ Full-text articles included journal articles found in peer-reviewed sources and included an abstract, introduction, methods, results, discussion and reference lists. ^2^ Research reports included short communications which did not meet full-text article inclusion criteria.

**Table 3:** Article type and outcome variables measured for the literature included in the scoping review.

	Type of Outcome Measured				
	Production	Tail-Biting	Blood Metabolites	Other	Total
Article Type ^1^					
Conference Proceedings	1	1	1	0	3
Full-Text Articles ^2^	38	81	12	45	176
Research Reports ^3^	2	6	0	2	10
Total	41	88	13	47	189

^1^ Possible for the same publication to have had more than one (1) outcome measured. Sixty-two (62) studies had more than one (1) outcome measured. ^2^ Full-text articles included journal articles found in peer-reviewed sources and included an abstract, introduction, methods, results, discussion and reference lists. ^3^ Research reports included short communications which did not meet full-text article inclusion criteria.

**Table 4:** Number of articles and the categories of tail-biting (TB) outcome measured.

# of Articles ^1^	Tail-Biting Outcome
4	None
59	Lesion frequency
77	Tail length/damage
10	Condemnations at slaughter
24	Other
174	Total

**^1^** Possible for the same publication to have had more than one (1) TB outcome measured. Fifty-nine (59) studies with more than one (1) TB outcome measured.

**Table 5:** Risk factors studied in the literature and the importance that was placed upon them in relation to controlling tail-biting (TB) successfully.

Charted Risk Factors	# of Articles ^1^	Important for TB Outcome	Unimportant for TB Outcome
Air Quality	18	5	13
Pig Flow	18	4	14
Diet	19	11	8
Feeding Frequency	22	14	8
Hygiene/Health	29	10	19
Housing/Flooring	46	31	15
Enrichment	62	48	14

**^1^** Possible for the same publication to have had more than one (1) risk factor studied and charted. Eighty-one (81) studies had more than one (1) risk factor charted.

**Table 6:** Intervention methods investigated and the number of successful and unsuccessful outcomes after the intervention had been applied.

Interventions	# of Articles ^1^	Successful	Unsuccessful
Straw Bedding/Rooting Substrate	22	17	5
Enrichment (Rope, Jute Sack, Wood, etc.)	12	8	4
Tail Length (Tail-Docking vs. Intact Tails)	11	9	2
Rearing Facility (Flooring, Enriched, etc.)	10	6	4
Stocking Density	8	4	4
Single-Sex Pens vs. Mixed-Sex Pens	4	1	3
Time that Enrichment Provided	4	1	3
Diet Type (Commercial vs. Specialty)	3	1	2
Pig Grouping (Litter mates, Tail-Biting Category)	3	0	3
Stress (ACTH Levels, Stimuli, R/I Test, etc.)	3	2	1
Feeding Frequency and Style	2	1	1
Advice from Veterinarians	2	2	0
Air Velocity and Direction	1	0	1
Identify Tail-Biters Early (Rope/Chew test)	1	0	1
Healthy vs. Ill Pigs	1	0	1
Number of Pigs per Feeder Space (Feeding Trough Density)	1	0	1
Tail-Biting Deterrent (Oil, Tar)	1	1	0
Teeth-Clipping	1	1	0

**^1^** Possible for the same publication to have had more than one (1) intervention applied. Fifteen (15) studies applied more than one (1) intervention.

## Supplementary Materials

The following are available online at https://www.mdpi.com/article/10.3390/ani11072002/s1, Supplementary File S1; Supplementary File S2: Citations used in the final scoping review. References [7,64,65,66,67,68,69,70,71,72,73,74,75,76,77,78,79,80,81,82,83,84,85,86,87,88,89,90,91,92,93,94,95,96,97,98,99,100,101,102,103,104,105,106,107,108,109,110,111,112,113,114,115,116,117,118,119,120,121,122,123,124,125,126,127,128,129,130,131,132,133,134] are cited in the supplementary materials.
