# Supplementary material for: Tail-Biting in Pigs: A Scoping Review
Source: Animals (Basel). 2021 Jul 5;11(7):2002. doi: 10.3390/ani11072002 (PMC8300120; doi:10.3390/ani11072002)
Supplement: Supplementary file 1 [file animals-11-02002-s001.zip › animals-1266481-SI/Sup file 1.pdf]

Supplementary material 1

Initial Protocol: May 31st, 2019

The risk factors associated with tail-biting in pigs and interventions researched to decrease or prevent this aberrant behaviour: a scoping review

Maggie Henry*[1], Hannah Jansen[1], Terri O'Sullivan[1], Lee Niel[1], Anna K. Shoveller[2], Robert M. Friendship[1]

*Corresponding Author

[1]Department of Population Medicine
[2]Department of Animal Biosciences

1. The Rationale

Tail-biting is a significant global welfare concern in commercial pig production. Tail-biting is a complex and difficult behaviour to both predict and prevent, due to environmental factors and the large variation in management styles of each producer and farm. In broad terminology, tail-biting is the oral manipulation of a pig's tail which may lead to damage and subsequent illness in the "bitten" pig. This aberrant behaviour appears to spread quickly among pen-mates and can grow into an outbreak situation which can then lead to significant loss of welfare for the affected pigs and loss of income for the producer.

A scoping review is a method of gathering literature, both published and non-published, in order to answer a research question. Scoping reviews are a fairly novel strategy for collecting data [Pham et. al 2014] that may help determine if a systematic review is justifiable [Arksey and O'Malley, 2005]. Scoping reviews can also help identify gaps in the literature as well as inform policy for industry stakeholders.

2. Objective

The primary objective of this scoping review is to identify and chart available literature on the risk factors associated with tail-biting in pigs, and to identify and describe interventions that have been trialed to decrease, prevent or eliminate tail-biting behaviour in commercial pig production systems. The secondary objective of this review is acknowledge and identify gaps between producers' and researchers' perceived risks of tail-biting in commercial pig production systems.

3. The review question

What are the main risk factors for tail-biting in pigs, which interventions have been researched since 1970, and what are the best intervention strategies to treat and prevent tail-biting?

4. Search strategy

Databases used [published literature]:

CABI, Web of Science, AGRICOLA

Database used [non-published literature]:

Search terms [SWINE, RISK, TAIL] were decided upon by identifying the relevant terms in the review question and by checking sensitivity of the terms and if they encompassed variations in terms that would be applicable to the scoping review.

Example: SWINE was used as a search term to verify that articles containing "pig", "pigs", "pork" and "*sus scrofa*" were all captured by the search term. It was concluded that SWINE was acceptable as an agreed upon term for the scoping review.

RISK and TAIL were both included in the search terms with an "*" at the terminus of each word to capture any words beginning with the root words "risk" and "tail" [example: risk factors, risks, tails].

Three search terms were used for both the published and un-published literature. The Boolean term AND was used between the three words. The search headings for each database was as follows:

SWINE **AND** TAIL* **AND** RISK*

5. Relevance Screening

Relevance screening will be undertaken by two independent reviewers and disagreements will be resolved by consensus. Relevance will be determined by looking at the title, summary and/or abstract of each article returned through the search strategy.

Eligibility criteria was decided upon to contain a date exclusion. Only articles published after and including 1970 were permitted to be screened by the reviewers. It was decided that this date was acceptable due substantial commercial pig production did not begin until the late 1960s, and therefore, a large body of research would not be available until the 1970s. All articles between January 1st, 1970 and May 31st, 2019 were considered acceptable, and were included in the inclusion criteria.

Language was also used as a search criterion. Only articles [both published and un-published] available in English were screened for relevance in this review. This was agreed upon as both reviewers only speak English and that a significant portion of relevant literature will either be published organically or have been translated into English.

The initial searches for Level 1 Screening yielded 212 articles from the 3 databases used for published literature [CABI: 137, Web of Science: 49 and AGRICOLA: 26]. The initial search for Level 1 Screening yielded 253 articles from the database for un-published or "grey" literature [AASV Information Library].

Agreed upon articles from both reviewers for Level 1 Screening yielded 47 published articles and 11 un-published or grey articles. The references from all agreed upon articles were screened to include any literature that may have been missed in the initial screening process. 61 articles were agreed upon by the two reviewers as meeting the relevant screening criteria for Level 1 analysis.

6. Data Extraction Form

The data extraction will be done based on the full article [both published and un-published] by two independent reviewers. Disagreements will be resolved by consensus.

Data Extraction Form

1. Does this study meet the Level 1 inclusion criteria?

[ ] Yes [ ] No

2. What country was the study conducted in?

[ ] Canada [ ] USA [ ] European Union [list country] [ ] Other [list country]

3. What is the citation type?

[ ] full text article [ ] full text thesis [ ] full text proceedings [e.g., short research paper cited in association with a conference or meeting] [ ] research/product report [ ] abstract only

4. What type of study is this? [choose one]

[ ] clinical trial [ ] challenge trial [list challenge] [ ] observational study [list study type: cohort, cross-sectional, case-control, etc.] [ ] none of the above

5. Year of publication _______

6. What was the sample size at the level at which the outcome was analyzed [collective total for all reported studies] [choose all that apply]

[ ] individual [n=___] [ ] pen/group/litter [n=___] [ ] herd [n=___

7. What was the production stage at the beginning of the study?

[ ] nursery [ ] grower [ ] finisher [ ] multiple stages [ ] gilt/sow

8. What was the production stage when the outcome was measured?

[ ] nursery [ ] grower [ ] finisher [ ] multiple stages [ ] gilt/sow

9. Was the population a commercial or a research population of pigs?

[ ] research [ ] commercial [ ] unspecified

10. Was there an intervention used? [ ] yes [ ] no

11. What intervention/management type risk factors were studied [choose all that apply]

[ ] air quality

[ ] hygiene

[ ] pig flow [list - mixing, moving, all-in-all-out, etc.]

[ ] diet type or format [list - pelleted feed, liquid feed, etc.]

[ ] housing or flooring [list - animal density, physical factors regarding feeder and water supply, indoor/outdoor rearing, slatted floor, solid floor, etc.]

[ ] feeding frequency [list - amount, schedule, etc.]

[ ] enrichment [list – toys, rooting material, etc.]

[ ] other [please specify]

12. What comparison group was used [choose all that apply]

[ ] no treatment [just usual or conventional practices]

[ ] placebo

[ ] exposure [please specify - no exposure, different level of exposure or treatment]

[ ] no comparison group used

13. What production outcome was measured? [choose all that apply]

[ ] none

[ ] tail-biting severity

[ ] performance [list - weight, ADG, feed intake, etc.]

[ ] cost/benefit analysis

[ ] other [list]

14. What tail-biting outcome[s] were measured [choose all that apply]

[ ] none

[ ] mortality

[ ] lesion frequency

[ ] tail length/tail damage

[ ] condemnations at slaughter

[ ] other [please specify]

15. Was the intervention successful?

[ ] yes [ ] no [ ] N/A

16. What successful intervention result was achieved, if any? [choose all that apply]

[ ] decreased frequency of tail-biting

[ ] decreased frequency of lesions

[ ] frequency of tail-biting remained constant

[ ] elimination of tail-biting

[ ] elimination of tail-docking

[ ] decreased/elimination of condemnations at slaughter

[ ] none

7. References

Arksey H. and O'Malley L. [2005] Scoping studies: towards a methodological framework. ***International Journal of Social Research Methodology.*** 8:1, 19-32

Pham M.T., Rajic A., Greig J.D., Sargeant J.M., Papadopoulos A. and McEwen S.A. [2014] A scoping review of scoping reviews: advancing the approach and enhancing the consistency. ***Research Synthesis Methods.*** 5: 371-385

Final Protocol: October 2019

Tail-biting in pigs: a scoping review

Maggie Henry*[1], Hannah Jansen[2], Maria Amezcua[1], Terri O'Sullivan[1], Lee Niel[1], Anna K. Shoveller[3] and Robert M. Friendship[1]

*Corresponding Author

[1]Department of Population Medicine
[2]Ontario Veterinary College
[3]Department of Animal Biosciences

1. The Rationale

Tail-biting is a significant global welfare concern in commercial pig production. Tail-biting is a complex and difficult behaviour to both predict and prevent, due to environmental factors and the large variation in management styles of each producer and farm. In broad terminology, tail-biting is the oral manipulation of a pig's tail which may lead to damage and subsequent illness in the "bitten" pig. This aberrant behaviour appears to spread quickly among pen-mates and can grow into an outbreak situation which can then lead to significant loss of welfare for the affected pigs and loss of income for the producer.

A scoping review is a method of gathering literature, both published and non-published, in order to answer a research question. Scoping reviews are a fairly novel strategy for collecting data [Pham et. al 2014] that may help determine if a systematic review is justifiable [Arksey and O'Malley, 2005]. Scoping reviews can also help identify gaps in the literature as well as inform policy for industry stakeholders.

2. Objective

The primary objective of this scoping review is to identify and chart available literature on the risk factors associated with tail-biting in pigs, and to identify and describe interventions that have been trialed to decrease, prevent or eliminate tail-biting behaviour in commercial pig production systems. The secondary objective of this review is acknowledge and identify gaps between producers' and researchers' perceived risks of tail-biting in commercial pig production systems.

3. The review question

What are the main risk factors for tail-biting in pigs, which intervention strategies have been researched since 1970, and what are the best intervention strategies to treat and prevent tail-biting?

4. Search strategy

Databases used [published literature]:

CABI, Web of Science, AGRICOLA

Database used [non-published literature]:

AASV Information Library

Search terms [SWINE, RISK, TAIL] were decided upon by identifying the relevant terms in the review question and by checking sensitivity of the terms and if they encompassed variations in terms that would be applicable to the scoping review.

Example: SWINE was used as a search term to verify that articles containing "pig", "pigs", "pork" and "*sus scrofa*" were all captured by the search term. It was concluded that SWINE was acceptable as an agreed upon term for the scoping review.

RISK and TAIL were both included in the search terms with an "*" at the terminus of each word to capture any words beginning with the root words "risk" and "tail" [example: risk factors, risks, tails].

Three search terms were used for both the published and un-published literature. The Boolean term AND was used between the three words. The search headings for each database was as follows:

SWINE AND TAIL* AND RISK*

5. Relevance Screening

Relevance screening will be undertaken by two independent reviewers and disagreements will be resolved by consensus. Relevance will be determined by looking at the title, summary and/or abstract of each article returned through the search strategy.

Eligibility criteria was decided upon to contain a date exclusion. Only articles published after and including 1970 were permitted to be screened by the reviewers. It was decided that this date was acceptable as substantial commercial pig production did not begin until the late 1960s, and therefore, a large body of research would not be available until the 1970s [REFERENCE]. All articles between January 1st, 1970 and May 31st, 2019 were considered acceptable, and were included in the inclusion criteria.

Language was also used as a search criterion. Only articles [both published and un-published] available in English were screened for relevance in this review. This was agreed upon as both reviewers only speak English and that a significant portion of relevant literature will either be published organically or have been translated into English.

The initial searches for Level 1 Screening yielded 212 articles from the 3 databases used for published literature [CABI: 137, Web of Science: 49 and AGRICOLA: 26]. The initial search for Level 1 Screening yielded 253 articles from the database for un-published or "grey" literature [AASV Information Library].

Agreed upon articles from both reviewers for Level 1 Screening yielded 47 published articles and 11 un-published or grey articles. The references from all agreed upon articles were screened to include any literature that may have been missed in the initial screening process. 61 articles were agreed upon by the two reviewers as meeting the relevant screening criteria for Level 1 analysis.

6. Data Extraction Form

The data extraction will be done based on the full article [both published and un-published] by two independent reviewers. Disagreements will be resolved by consensus.

Data Extraction Form

1. Should this article be included in the final Scoping Review

[ ] yes [ ] no

2. Does this study meet the Level 1 inclusion criteria?

[ ] yes [ ] no

3. Year of publication _______

4. What country was the study conducted in?

[ ] Canada [ ] USA [ ] European Union [list country] [ ] Other [list country]

5. What is the citation type?

[ ] full text article [ ] full text thesis [ ] full text proceedings [e.g., short research paper cited in association with a conference or meeting] [ ] research/product report [ ] abstract only

6. What type of study is this? [choose one]

[ ] clinical trial [ ] observational study [list study type: cohort, cross-sectional, case-control, etc.] [ ] none of the above

7. What was the sample size at the level at which the outcome was analyzed [choose all that apply]

[ ] individual [n=___] [ ] pen/group/litter [n=___] [ ] herd [n=___]

8. What was the production stage at the beginning of the study?

[ ] birth/pre-weaning [ ] nursery [ ] grower [ ] finisher [ ] gilt/sow

9. What was the production stage when the outcome was measured?

[ ] birth/pre-weaning [ ] nursery [ ] grower [ ] finisher [ ] gilt/sow

10. Was the population a research or commercial population of pigs?

[ ] research [ ] commercial [ ] unspecified

11. Was there an intervention used?

[ ] yes [ ] no

12. What was the intervention used?

[ ] none [ ] please list

13. If an intervention was used, at what level was it implemented at?

[ ] individual [ ] group [ ] pen [ ] herd [ ] other [please list] [ ] N/A

14. What risk factors were studied [choose all that apply]

[ ] air quality

[ ] hygiene/health

[ ] pig flow [list - mixing, moving, all-in-all-out, etc.]

[ ] housing or flooring [list - animal density, physical factors regarding feeder and water supply, indoor/outdoor rearing, slatted floor, solid floor, etc.]

[ ] diet type or format [list - pelleted feed, liquid feed, etc.]

[ ] feeding frequency [list - amount, schedule, etc.]

[ ] enrichment [list – toys, rooting material, etc.]

[ ] other [please specify]

15. What comparison group was used [choose all that apply]

[ ] usual or conventional practices for that system

[ ] exposure [please specify - no exposure, different level of exposure or treatment]

[ ] no comparison group used

16. What type of outcome was measured? [choose all that apply]

[ ] none

[ ] production

[ ] tail-biting

[ ] gut morphology

[ ] blood metabolites

[ ] other [list]

17. What tail-biting outcome[s] were measured [choose all that apply]

[ ] none

[ ] lesion frequency

[ ] tail length/tail damage

[ ] condemnations at slaughter

[ ] other [please specify]

18. Was the intervention successful?

[ ] yes [ ] no [ ] N/A

19. What successful intervention result was achieved, if any? [choose all that apply]

[ ] decreased frequency of tail-biting

[ ] decreased frequency of lesions

[ ] frequency of tail-biting remained constant

[ ] elimination of tail-biting

[ ] elimination of tail-docking

[ ] decreased/elimination of condemnations at slaughter

[ ] none

20. Which risk factors from the study were found to be important? [please list]

[ ] please list [ ] none

21. Which risk factors from the study were found not to be important? [please list]

[ ] please list [ ] none

7. References

Arksey H. and O'Malley L. [2005] Scoping studies: towards a methodological framework. *International Journal of Social Research Methodology.* 8:1, 19-32.

Pham M.T., Rajic A., Greig J.D., Sargeant J.M., Papadopoulos A. and McEwen S.A. [2014] A scoping review of scoping reviews: advancing the approach and enhancing the consistency. *Research Synthesis Methods.* 5: 371-385.
