# Supplementary material for: Tail-Biting in Pigs: A Scoping Review
Source: Animals (Basel). 2021 Jul 5;11(7):2002. doi: 10.3390/ani11072002 (PMC8300120; doi:10.3390/ani11072002)
Supplement: Supplementary file 1 [file animals-11-02002-s001.zip › animals-1266481-SI/Sup file 2_revised.pdf]

Supplementary material 2– Citations used in the final scoping review.

64. Baker, S.; Hough, S.; Jablonski, E.; Angulo, J.; Nemechek, E. Development of a scoring system to determine the prevalence, severity,and associated risk factors of tail-biting and lameness. 50th Annual Meeting of the American Association of Swine Veterinarians, Orlando, U.S.A., March 2019; 103-104.

65. Beattie, V.E.; Walker, N.; Sneddon, I.A. An investigation of the effect of environmental enrichment and space allowance on the behaviour and production of growing pigs. *Appl Anim Behav Sci.* 1996, *48*, 151-158.

66. Beattie, V.E.; Breuer, K.; O'Connell, N.E.; Sneddon, I.A.; Mercer, J.T.; Rance, K.A.; Sutcliffe, M.E.M.; Edwards, S.A. Factors identifying pigs predisposed to tail biting. *Anim Sci*. 2005, *80*, 307-312.

67. Blackshaw, J.K. Some behavioural deviations in weaned domestic pigs: persistent inguinal nose thrusting, and tail and ear biting. *Anim Prod.* 1981, *33*, 325-332.

68. Bodin, L.; Algers, B.; Andersson, M.; Olsson, A.C.; Botermans, J. The amount of straw for growing-finishing pigs considering the reduction of time spent in manipulative behavior. *SOJ Vet Sci.* 2015, *1*, 1-6.

69. Bolhuis, J.E.; Schouten, W.G.P.; Schrama, J.W.; Wiegant, V.M. Behavioural development of pigs with different coping characteristics in barren and substrate-enriched housing conditions. *Appl Anim Behav Sci.* 2005, *93*, 213-228.

70. Bracke, M.B.M.; De Lauwere, C.C.; Wind, S.M.M.; Zonerland, J.J. Attitudes of Dutch Pig Farmers Towards Tail Biting and Tail Docking. *J Agric Environ Ethics.* 2013, *26*, 847-868.

71. Bracke, M.B.M. Rope test may indicate efficacy of tail-biting treatments in growing pigs. *Anim Welf.* 2009, *18*, 263-266.

72. Cagienard, A.; Regula, G.; Danuser, J. The impact of different housing systems on health and welfare of grower and finisher pigs in Switzerland. *Prev Vet Med.* 2005, *68*, 49-61.

73. Calderón Díaz, J.A.; Boyle, L.A.; Diana, A.; Leonard, F.C.; Moriarty, J.P.; McElroy, M.C.; McGettrick, S.; Kelliher, D.; García Manzanilla, E. Early life indicators predict mortality, illness, reduced welfare and carcass characteristics in finisher pigs. *Prev Vet Med.* 2017, *146*, 94-102.

74. Camerlink, I.; Ursinus, W.W.; Bijma, P.; Kemp, B.; Bolhuis, J.E. Indirect genetic effects for growth rate in domestic pigs alter aggressive and manipulative biting behaviour. *Behav Genet.* 2015, *45*, 117-126.

75. Chou, J-Y.; D'Eath, R.B.; Sandercock, D.A.; Waran, N.; Haigh, A.; O'Driscoll, K. Use of different wood types as environmental enrichment to manage tail biting in docked pigs in a commercial fully-slatted system. *Livest Sci.* 2018, *213*, 19-27.

76. Cornale, P.; Macchi, E.; Miretti, S.; Renna, M.; Lussiana, C.; Perona, G.; Mimosi, A. Effects of stocking density and environmental enrichment on behavior and fecal corticosteroid levels of pigs under commercial farm conditions. *J Vet Behav.* 2015, *10*, 569-576.

77. Cox, L.N.; Cooper, J.J. Observations on the pre- and post-weaning behaviour of piglets reared in commercial indoor and outdoor environments. *Anim Sci.* 2001, *72*, 75-86.

78. D'Eath, R.B.; Jack, M.; Futro, A.; Talbot, D.; Zhu, Q.; Barclay, D.; Baxter, E.M. Automatic early warning of tail biting in pigs: 3D cameras can detect lowered tail posture before an outbreak. *PLOS One* 2018, *13*, 1-18. https://doi.org/10.1371/journal.pone.0194524

79. Day, J.E.L.; Burfoot, A.; Docking, C.M.; Whittaker, X.; Spoolder, H.A.M.; Edwards, S.A. The effects of prior experience of straw and the level of straw provision on the behaviour of growing pigs. *Appl Anim Behav Sci.* 2002, *76*, 189-202.

80. De Briyne, N.; Berg, C.; Blaha, T.; Palzer, A.; Temple, D. Phasing out pig tail docking in the EU - present state, challenges and possibilities. *Porc Health Manag.* 2018, *4*, 1-9.

81. Dybkjær, L. The identification of behavioural indicators of 'stress' in early weaned piglets. *Appl Anim Behav Sci.* 1992, *35*, 135-147.

82. Edwards, S.A.; Scott, K.; Armstrong, D.; Taylor, L.; Gill, B.P.; Chennells, D.J.; Hunt, B. Finishing pig systems: health and welfare in straw-bedded or slatted housing. *Pig J.* 2005, *56*, 174-178.

83. Ewbank, R. Abnormal behaviour and pig nutrition. An unsuccessful attempt to induce tail biting by feeding a high energy, low fibre vegetable protein ration. *Br Vet J.* 1973, *129*, 366-369.

84. Fu, L.; Zhou, B.; Li, H.; Schinckel, A.P.; Liang, T.; Chu, Q.; Li, Y.; Xu, F. Teeth clipping, tail docking and toy enrichment affect physiological indicators, behaviour and lesions of weaned pigs after re-location and mixing. *Livest Sci.* 2018, *212*, 137-142.

85. Goossens, X.; Sobry, L.; Ödberg, F.; Tuyttens, F.; Maes, D.; De Smet, S.; Nevens, F.; Opsomer, G.; Lommelen, F.; Geers, R. A population-based on-farm evaluation protocol for comparing the welfare of pigs between farms. *Anim Welf.* 2008, *17*, 35-41.

86. Guy, J.H.; Rowlinson, P.; Chadwick, J.P.; Ellis, M. Behaviour of two genotypes of growing-finishing pig in three different housing systems. *Appl Anim Behav Sci.* 2002, *75*, 193-206.

87. Hunter, E.J.; Jones, T.A.; Guise, H.J.; Penny, R.H.C.; Hoste, S. Tail biting in pigs 1: The prevalence at six UK abattoirs and the relationship of tail biting with docking, sex and other carcass damage. *The Pig Journal* 1999, *43*, 18-32.

88. Jensen, MB.; Studnitz, M.; Pedersen, L.J. The effect of type of rooting material and space allowance on exploration and abnormal behaviour in growing pigs. *Appl Anim Behav Sci.* 2010, *123*, 87-92.

89. Kallio, P.A.; Janczak, A.M.; Valros, A.E.; Edwards, S.A.; Heinonen, M. Case control study on environmental, nutritional and management-based risk factors for tail-biting in long-tailed pigs. *Anim Welf*. 2018, *27*, 21-34.

90. Kritas, S.K.; Morrison, R.B. An observational study on tail biting in commercial grower-finisher barns. *J Swine Health Prod.* 2004, *12*,17-22.

91. Lahrmann, H.P.; Hansen, C.F.; D´Eath, R.B.; Busch, M.E.; Nielsen, J.P.; Forkman, B. Early intervention with enrichment can prevent tail biting outbreaks in weaner pigs. *Livest Sci.* 2018, *214*, 272-277.

92. Lahrmann, H.P.; Busch, M.E.; D'Eath, R.B.; Forkman, B.; Hansen, C.F. More tail lesions among undocked than tail docked pigs in a conventional herd. *Animal* 2017, *11*, 1825-1831.

93. Lahrmann, H.P.; Hansen, C.F.; D'Eath, R.; Busch, M.E.; Forkman, B. Tail posture predicts tail biting outbreaks at pen level in weaner pigs. *Appl Anim Behav Sci*. 2018, *200*, 29-35.

94. Lahrmann, H.P.; Oxholm, L.C.; Steinmetz, H.; Nielsen, M.B.F.; D'Eath, R.B. The effect of long or chopped straw on pig behaviour. *Animal* 2015, *9*, 862-870.

95. Larsen, M.L.V.; Andersen, H.M.-L.; Pedersen, L.J. Which is the most preventive measure against tail damage in finisher pigs: tail docking, straw provision or lowered stocking density? *Animal* 2018, *12*, 1260-1267.

96. Laskoski, F.; Faccin, J.E.G.; Vier, C.M.; Gonçalves, M.A.D.; Orlando, U.A.D.; Kummer, R.; Mellagi, A.P.G.; Bernardi, M.L.; Wentz, I.; Bortolozzo, F.P. Effects of pigs per feeder hole and group size on feed intake onset, growth performance, and ear and tail lesions in nursery pigs with consistent space allowance. *J Swine Health Prod.* 2019, *27*, 12-18.

97. McIntyre, J.; Edwards, S.A. An investigation into the effect of different protein and energy intakes on model tail chewing behaviour of growing pigs. *Appl Anim Behav Sci.* 2002, *77*, 93-104.

98. McKinnon, A.J.; Edwards, S.A.; Stephens, D.B.; Walters, D.E. Behaviour of groups of weaner pigs in three different housing systems. *Br Vet J.* 1989, *145*, 367-372.

99. Munsterhjelm, C.; Nordgreen, J.; Aae, F.; Heinonen, M.; Olstad, K.; Aasmundstad, T.; Janczak, A.M.; Valros, A. To be blamed or pitied? The effect of illness on social behavior, cytokine levels and feed intake in undocked boars. *Physiol Behav.* 2017, *179*, 298-307.

100. Palander, P.A.; Heinonen, M.; Simpura, I.; Edwards, S.A.; Valros, A.E. Jejunal morphology and blood metabolites in tail biting, victim and control pigs. *Animal* 2013, *7*, 1523-1531.

101. Pandolfi, F.; Kyriazakis, I.; Stoddart, K.; Wainwright, N.; Edwards, S.A. The "Real Welfare" scheme: Identification of risk and protective factors for welfare outcomes in commercial pig farms in the UK. *Prev Vet Med.* 2017, *146*, 34-43.

102. Penny, R.H.C. Tail-biting in pigs a sex frequency between boars and gilts. *Vet Rec.* 1981, *108*, 35.

103. Petersen, V.; Simonsen, H.B.; Lawson, L.G. The effect of environmental stimulation on the development of behaviour in pigs. *Appl Anim Behav Sci.* 1995. *45*, 215-224.

104. Sällvik, K.; Walberg, K. The effects of air velocity and temperature on the behaviour and growth of pigs. *J agric Engng Res.* 1984, *30*, 305-312.

105. Schmolke, S.A.; Li, Y.Z.; Gonyou, H.W. Effect of group size on performance of growing-finishing pigs. *J Anim Sci.* 2003, *81*, 874-878.

106. Scott, K; Chennells, DJ; Armstrong, D; Taylor, L; Gill, BP; Edwards, SA. The welfare of finishing pigs under different housing and feeding systems: liquid versus dry feeding in fully-slatted and straw-based housing. *Anim Welf.* 2007, *16*, 53-62.

107. Smulders, D.; Hautekiet, V.; Verbeke, G.; Geers, R. Tail and ear biting lesions in pigs: an epidemiological study. *Anim Welf.* 2008, *17*, 61-69.

108. Statham, P.; Green, L.; Bichard, M.; Mendl, M. Predicting tail-biting from behaviour of pigs prior to outbreaks. *Appl Anim Behav Sci.* 2009, *121*, 157-164.

109. Sutherland, M.A.; Bryer, P.J.; Krebs, N.; McGlone, J.J. The effect of method of tail docking on tail-biting behaviour and welfare of pigs. *Anim Welf.* 2009, *18*, 561-570.

110. Telkänranta, H.; Bracke, M.B.M.; Valros, A. Fresh wood reduces tail and ear biting and increases exploratory behaviour in finishing pigs. *Appl Anim Behav Sci.* 2014, *161*, 51-59.

111. Temple, D.; Courboulay, V.; Velarde, A.; Dalmau, A.; Manteca, X. The welfare of growing pigs in five different production systems in France and Spain: assessment of health. *Anim Welf.* 2012, *21*, 257-271.

112. Ursinus, W.W.; Wijnen, H.J.; Bartels, A.C.; Dijvesteijn, N.; van Reenen, C.G.; Bolhuis, J.E. Damaging biting behaviors in intensively kept rearing gilts: The effect of jute sacks and relations with production characteristics *J Anim Sci.* 2014, *92*, 5193-5202.

113. Ursinus, W.W.; Van Reenen, C.G.; Kemp, B.; Bolhuis, J.E. Tail biting behaviour and tail damage in pigs and the relationship with general behaviour: Predicting the inevitable? *Appl Anim Behav Sci.* 2014*, 156*, 22-36.

114. Ursinus, W.W.; Van Reenen, C.G.; Reimert, I.; Bolhuis, J.E. Tail Biting in Pigs: Blood Serotonin and Fearfulness as Pieces of the Puzzle? *PLOS One* 2014, *9*, 1-14. https://doi.org/10.1371/journal.pone.0107040

115. Valros, A.; Palander, P.; Heinonen, M.; Munsterhjelm, C.; Brunberg, E.; Keeling, L.; Piepponen, P. Evidence for a link between tail biting and central monoamine metabolism in pigs (Sus scrofa domestica). *Physiol Behav*. 2015, *143*, 151-157.

116. Valros, A.; Munsterhjelm, C.; Puolanne, E.; Ruusunen, M.; Heinonen, M.; Peltoniemi, O.A.T.; Pösö, A.R. Physiological indicators of stress and meat and carcass characteristics in tail bitten slaughter pigs. *Acta Vet Scand.* 2013, *55*, 1-8.

117. Van de Weerd, H.A.; Docking, C.M.; Day, J.E.L.; Breuer, K.; Edwards, S.A. Effects of species-relevant environmental enrichment on the behaviour and productivity of finishing pigs. *Appl Anim Behav Sci.* 2006, *99*, 230-247.

118. Van de Weerd, H.A.; Docking, C.M.; Day, J.E.L.; Edwards, S.A. The development of harmful social behaviour in pigs with intact tails and different enrichment backgrounds in two housing systems. *Anim Sci.* 2005, *80*, 289-298.

119. van Staaveren, N.; Teixeira, D.L.; Hanlon, A.; Boyle, L.A. Pig carcass tail lesions: the influence of record keeping through an advisory service and the relationship with farm performance parameters. *Animal* 2017, *11*, 140-146.

120. Veit, C.; Traulsen, I.; Hasler, M.; Tölle, K-H.; Burfeind, O.; grosse Beilage, E.; Krieter, J. Influence of raw material on the occurrence of tail-biting in undocked pigs. *Livest Sci.* 2016, *191*, 125-131.

121. Veit, C.; Büttner, K.; Traulsen, I.; Gertz, M.; Hasler, M.; Burfeind, O.; grosse Beilage, E.; Krieter, J. The effect of mixing piglets after weaning on the occurrence of tail-biting during rearing. *Livest Sci.* 2017, *201*, 70-73.

122. Walker, P.K.; Bilkei, G. Tail-biting in outdoor pig production. *Vet J.* 2006, *171*, 367-369.

123. Wallenbeck, A.; Keeling, L.J. Using data from electronic feeders on visit frequency and feed consumption to indicate tail biting outbreaks in commercial pig production1. *J Anim Sci.* 2013, *91*, 2879-2884.

124. Wallgren, T.; Westin, R.; Gunnarsson, S. A survey of straw use and tail biting in Swedish pig farms rearing undocked pigs. *Acta Vet Scand.* 2016, *58*, 1-11.

125. Wallgren, T.; Larsen, A.; Lundeheim, N.; Westin, R.; Gunnarsson, S. Implication and impact of straw provision on behaviour, lesions and pen hygiene on commercial farms rearing undocked pigs. *Appl Anim Behav Sci.* 2019, *210*, 26-37.

126. Wallgren, T.; Larsen, A.; Gunnarsson, S. Tail posture as an indicator of tail biting in undocked finishing pigs. *Animals* 2019, *9*, 1-11.

127. Wedin, M.; Baxter, E.M.; Jack, M.; Futro, A.; D'Eath, R.B. Early indicators of tail biting outbreaks in pigs. *Appl Anim Behav Sci.* 2018, *208*, 7-13.

128. Wilson, K.; Zanella, R.; Ventura, C.; Johansen, H.L.: Framstad, T.; Janczak, A.; Zanella, A.J.; Neibergs, H.L. Identification of chromosomal locations associated with tail biting and being a victim of tail-biting behaviour in the domestic pig (Sus scrofa domesticus). *J Appl Genetics* 2012, *53*, 449-456.

129. Zonderland, J.J.; Schepers, F.; Bracke, M.B.M.; den Hartog, L.A.; Kemp, B.; Spoolder, H.A.M. Characteristics of biter and victim piglets apparent before a tail-biting outbreak. *Animal* 2011, *5*, 767-775.

130. Zonderland, J.J.; Bracke, M.B.M.; den Hartog, L.A.; Kemp, B.; Spoolder, H.A.M. Gender effects on tail damage development in single- or mixed-sex groups of weaned piglets. *Livest Sci.* 2010, *129*, 151-158.

131. Zonderland, J.J.; Kemp, B.; Bracke, M.B.M.; den Hartog, L.A.; Spoolder, H.A.M. Individual piglets' contribution to the development of tail biting. *Animal* 2011, *5*, 601-607.

132. Zonderland, J.J.; Wolthuis-Fillerup, M.; van Reenen, C.G.; Bracke, M.B.M.; Kemp, B.; den Hartog, L.A.; Spoolder, H.A.M. Prevention and treatment of tail biting in weaned piglets. *Appl Anim Behav Sci.* 2008, *110*, 269-281.

133. Zonderland, J.J.; van Riel, J.W.; Bracke, M.B.M.; Kemp, B.; den Hartog, L.A.; Spoolder, H.A.M. Tail posture predicts tail damage among weaned piglets. *Appl Anim Behav Sci.* 2009, *121*, 165-170.
